# Transcriptional characterization of conjunctival melanoma identifies the cellular tumor microenvironment and prognostic gene signatures

**DOI:** 10.1038/s41598-020-72864-0

**Published:** 2020-10-12

**Authors:** Julian Wolf, Claudia Auw-Haedrich, Anja Schlecht, Stefaniya Boneva, Hans Mittelviefhaus, Thabo Lapp, Hansjürgen Agostini, Thomas Reinhard, Günther Schlunck, Clemens A. K. Lange

**Affiliations:** Eye Center, Medical Center, Faculty of Medicine, University of Freiburg, Killianstrasse 5, 79106 Freiburg, Germany

**Keywords:** Prognostic markers, Cancer genomics, Eye cancer, Tumour biomarkers, Cancer microenvironment

## Abstract

This study characterizes the transcriptome and the cellular tumor microenvironment (TME) of conjunctival melanoma (CM) and identifies prognostically relevant biomarkers. 12 formalin-fixed and paraffin-embedded CM were analyzed by MACE RNA sequencing, including six cases each with good or poor clinical outcome, the latter being defined by local recurrence and/or systemic metastases. Eight healthy conjunctival specimens served as controls. The TME of CM, as determined by bioinformatic cell type enrichment analysis, was characterized by the enrichment of melanocytes, pericytes and especially various immune cell types, such as plasmacytoid dendritic cells, natural killer T cells, B cells and mast cells. Differentially expressed genes between CM and control were mainly involved in inhibition of apoptosis, proteolysis and response to growth factors. *POU3F3*,* BIRC5* and *7* were among the top expressed genes associated with inhibition of apoptosis. 20 genes, among them *CENPK*, *INHA*, *USP33*,* CASP3*,* SNORA73B*,* AAR2*,* SNRNP48* and *GPN1*, were identified as prognostically relevant factors reaching high classification accuracy (area under the curve: 1.0). The present study provides new insights into the TME and the transcriptional profile of CM and additionally identifies new prognostic biomarkers. These results add new diagnostic tools and may lead to new options of targeted therapy for CM.

## Introduction

Conjunctival melanoma (CM) is a rare but potentially life-threatening tumor of the ocular surface associated with systemic metastasis within 10 years in 18–26% and a 10 year survival rate of 41–78%^1^. Age-adjusted incidence is estimated at 0.55 per million per year in the United States with a tendency to increase from 0.26 in 1973 to 0.55 per million per year in 1999^2^ accounting for 0.24% of all melanoma^3^. The majority of CM originates from primary acquired melanosis (57–76%), whereas a smaller proportion of CM develops de novo (16–25%) or from a pre-existing nevus (1–6%)^1,4^. Confirmed risk factors are the localization, with worse prognosis being reported for fornices, caruncle, plica semilunaris, palpebral conjunctiva and lid margins^5^, the tumor thickness^5^, the TNM stage^6^ and de novo CM^1^. There is evidence that UV radiation contributes to the development of CM^7^, although this factor is still debated^8^. It has also been shown that melanization stimulates HIF-1α expression of melanoma cells^9^, affects clinical outcome of patients with cutaneous and uveal melanoma^10,11^ and modifies the sensitivity to anticancer treatment, including radio- and immunotherapy^12–14^. The expression of progesterone and estrogen receptors on conjunctival melanomas could explain hormone-dependent changes in tumor morphology^15^. Based on the genetic profile, CM seems to be a distinct subset of melanoma, which is more similar to cutaneous melanoma than mucosal melanoma and is significantly different from uveal melanoma^16^. It is well known that CM is often associated with mutations in *BRAF*, representing a potential therapeutic target^17^. Nevertheless, little is known about the transcriptome and the cellular tumor microenvironment (TME) of CM, which could provide additional information about the pathways, molecular mechanisms and cell types involved in the pathogenesis of CM and thus define potentially new diagnostic and therapeutic targets.

**Figure 1:**
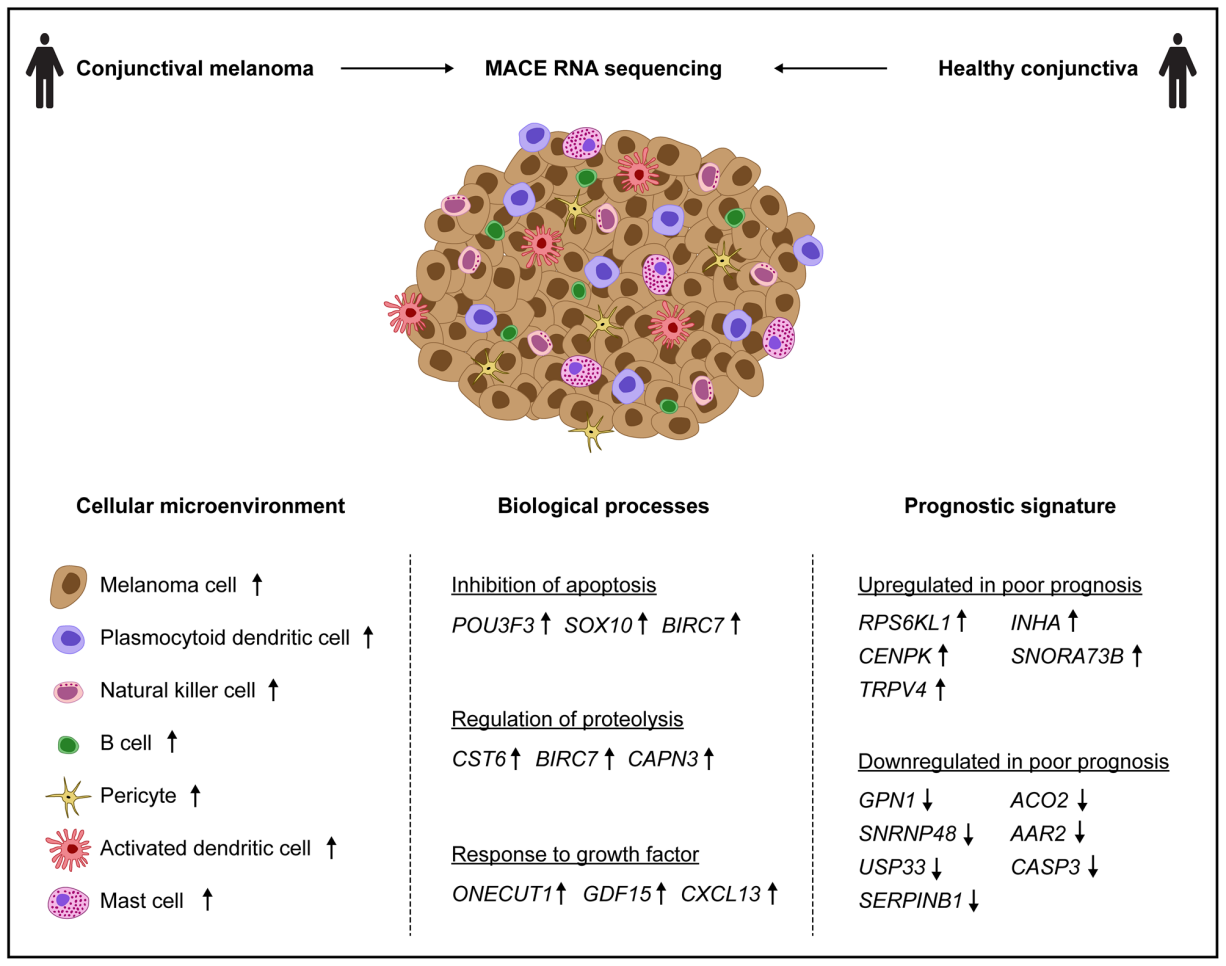
Study overview. The study characterizes the transcriptional profile of conjunctival melanoma compared to healthy conjunctiva, provides new insights into the cellular tumor microenvironment and identifies a prognostic signature classifying clinical outcome of conjunctival melanoma.

This study characterizes the cellular tumor microenvironment and provides a transcriptional profile of CM compared to healthy conjunctiva and analyzes the transcriptional differences between CM with poor and good clinical outcome (Fig. 1). The results of this study add new diagnostic and prognostic tools and may lead to new options of targeted therapy for CM.

## Results

### Patient characteristics

A total of 20 conjunctival samples from 20 patients were included in this study. Histological analysis confirmed CM in 12 patients and healthy conjunctiva in eight patients. Patient characteristics are summarized in Table 1. Mean age in the melanoma and the control group was 58.9 (range: 27.3–85.3) and 55.7 years (range: 43.0—69.0), respectively (*p* = 0.692). There were three male and nine female patients in the melanoma group and six male and two female patients in the control group, respectively (*p* = 0.081). Pathologic T (pT) categories according to the American Joint Committee on Cancer (AJCC) Cancer Staging Manual (eighth edition)^18^ were pT1a in 9 (75.0%), pT1b in 1 (8.3%) and pT3b in 2 (16.6%) cases. Mean tumor thickness was 944.1 µm (range: 199.0 µm—2720.0 µm). Nine (75.0%) cases were located at the bulbar conjunctiva and one (8.3%) each at the fornix, tarsal conjunctiva and limbus. There were four patients with local recurrence (three cases with different localization between primary tumor and recurrence), one patient with local recurrence and systemic metastasis and one patient with systemic metastasis, which were included in the poor prognosis group. The mean follow-up time in the melanoma group was 7.1 years (min: 2.2, max: 16.7) (see Table 1).

**Table 1:** Patient characteristics.

Group	Conjunctival melanoma	Healthy conjunctiva	*p*
n	12	8	–
Age at surgery (years)	58.9 (27.3–85.3)	55.7 (43.0–69.0)	0.692
Sex			0.081
Male	3 (25.0%)	6 (75.0%)	
Female	9 (75.0%)	2 (25.0%)	
pT-category		–	–
pT1a	9 (75.0%)		
pT1b	1 (8.3%)		
pT3b	2 (16.6%)		
Tumor-thickness (µm)	944.1 (199.0–2720.0)	–	–
Localization		–	–
Bulbar conjunctiva	9 (75.0%)		
Fornix	1 (8.3%)		
Tarsal conjunctiva	1 (8.3%)		
Limbus	1 (8.3%)		
Local recurrence		–	–
Different localization	3		
Similar localization	1		
Systemic metastasis	1	–	–
Local recurrence and systemic metastasis	1	–	–
Follow-up (years)	7.1 (2.2–16.7)	–	–

pT-classification according to the eighth edition of the AJCC conjunctival melanoma staging system^18^. Data is shown as mean (range) or as absolute (relative) numbers.

### Unsupervised transcriptomic analysis

**Figure 2:**
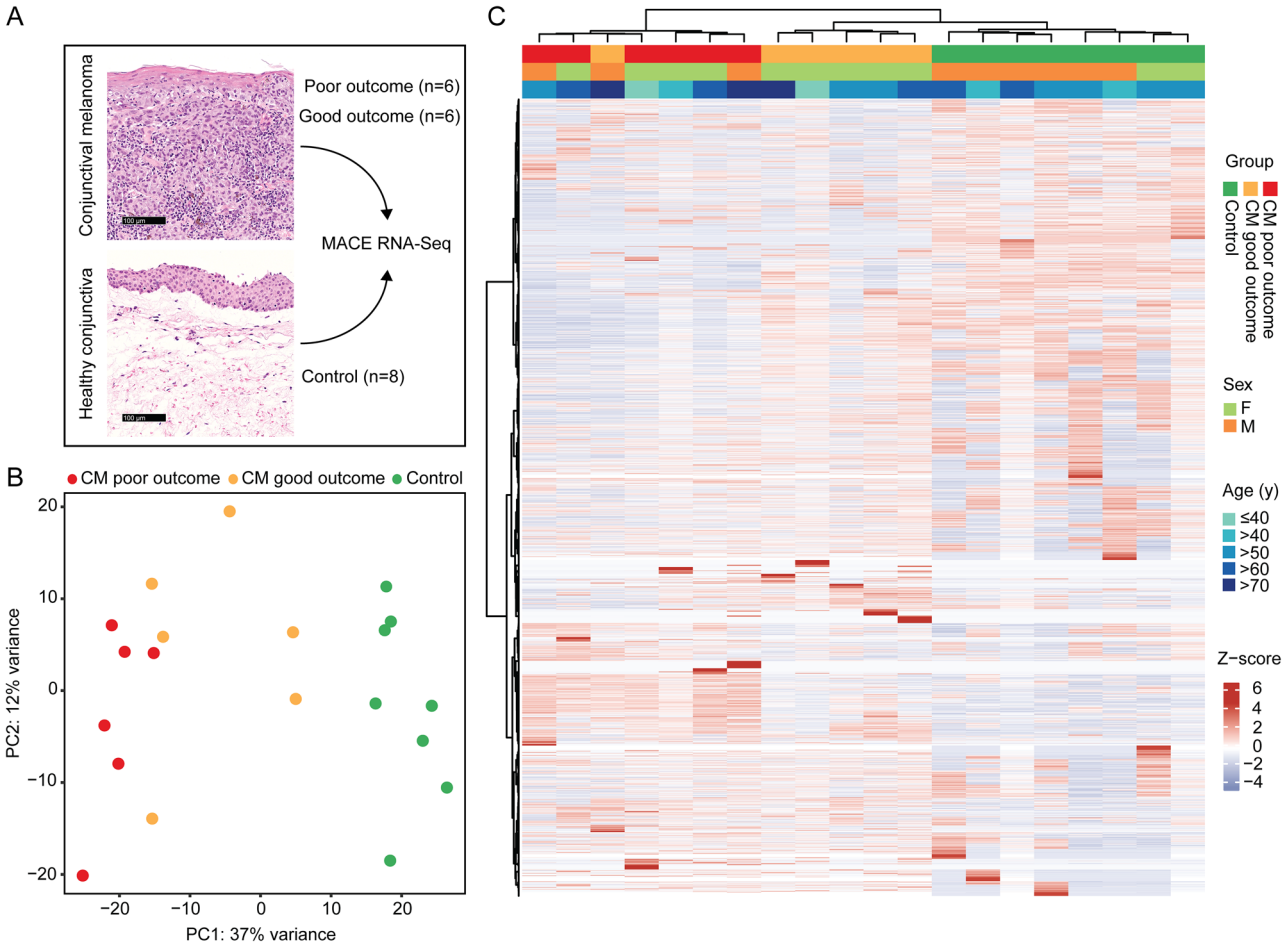
Unsupervised cluster analysis of twelve conjunctival melanoma (CM, six each with good or poor outcome) and eight normal conjunctival samples. Clinical outcome was classified based on the presence or absence of local recurrence and/or systemic metastases, respectively, with a follow up of at least 24 months. (**A**) Representative histological specimens of melanoma and normal conjunctiva as well as an overview of the study design. (**B**) Principle component analysis. (**C**) Unsupervised heatmap: basic demographic and clinical data are shown above. Each column represents one sample (red: melanoma with poor outcome, orange: melanoma with good outcome and green: healthy conjunctiva) and each row one expressed gene. There were 20.189 genes with at least one raw read. Unsupervised clustering was performed for both the rows and the columns (see dendrogram). The z-score represents a gene’s expression in relation to its mean expression by standard deviation units (red: upregulation, blue: downregulation). CM: melanoma, PC: principle component.

Unsupervised analysis revealed distinct differences in the transcriptome of CM when compared to normal conjunctiva. In addition, melanoma with poor and good clinical outcome also differed significantly with regard to their transcriptional profile (see PCA and unsupervised heatmap, Fig. 2).

### Cellular tumor microenvironment of conjunctival melanoma

**Figure 3:**
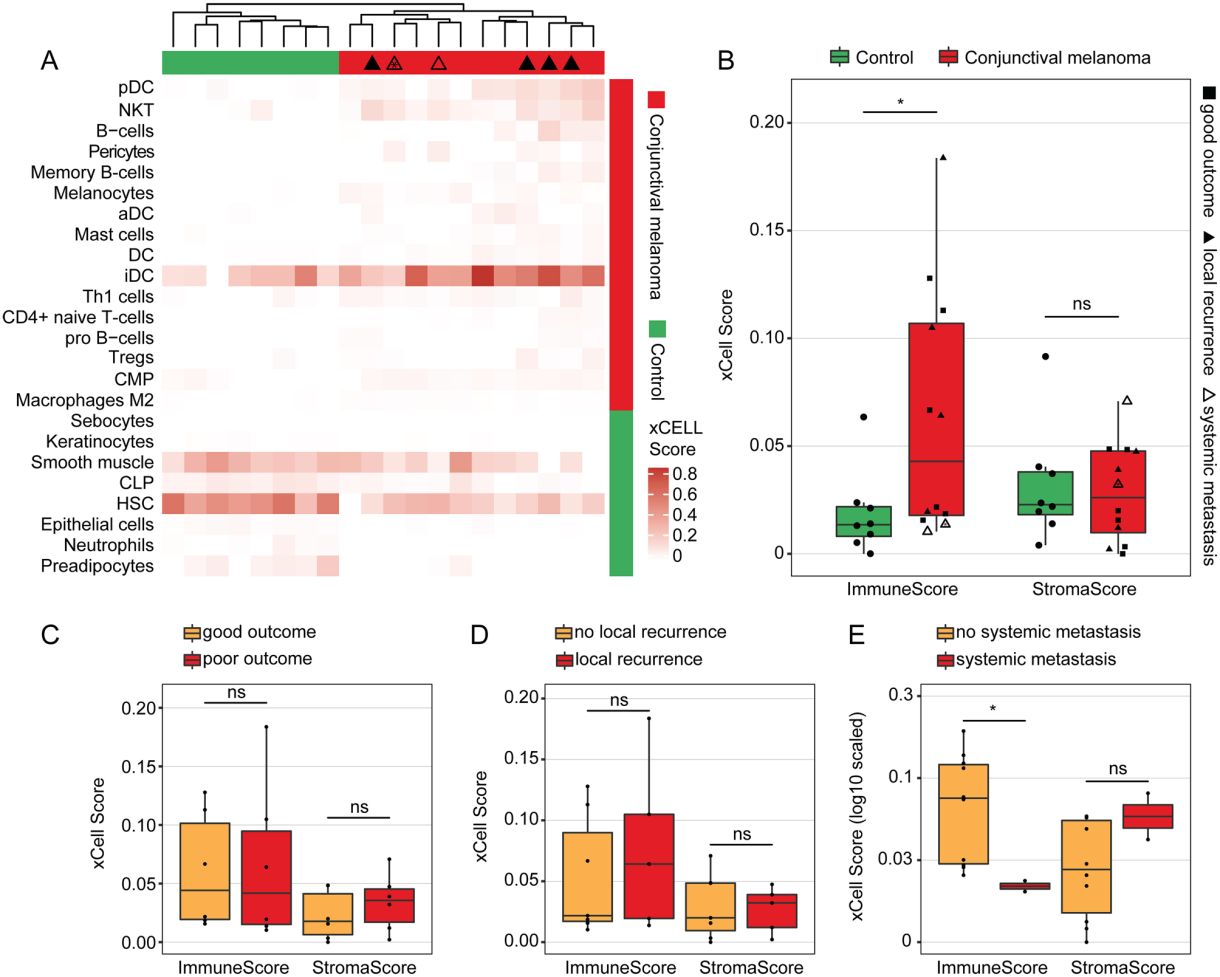
Cellular tumor microenvironment of conjunctival melanoma characterized by cell type enrichment analysis using xCell. The tool uses gene expression profiles of 64 immune and stromal cell types to calculate cell type enrichment scores. (**A**) Heatmap illustrating xCell enrichment scores of 24 of the 64 cell types which were significantly enriched in melanoma compared to normal conjunctiva (*p* < 0.05, Mann–Whitney U test). 16 cell types were up- and 8 cell types were downregulated in melanoma (see annotation on the right of the heatmap). Each row represents one cell type, each column represents one sample. Rows are ordered according to the fold change of mean enrichment scores for melanoma and normal conjunctiva, respectively. Columns are clustered according to similarities in xCell enrichment scores (see dendrogram). Samples with local recurrence and/or systemic metastasis are labeled (see legend in B, *: local recurrence and systemic metastasis). pDC: plasmacytoid dendritic cell, NKT: natural killer T cell, aDC: activated dendritic cell, DC: dendritic cell, iDC: immature dendritic cells, Th1: type 1 T-helper cells, Tregs: regulatory T cells, CMP: common myeloid progenitor, CLP: common lymphoid progenitor, HSC: hematopoietic stem cell. (**B**) Boxplots of the xCell immune and stroma score between melanoma and healthy conjunctiva. Each symbol represents one sample and the shape represents the prognostic groups also shown in (**C**–**E**). (**C–E**): boxplots of the xCell immune and stroma score between melanoma with poor and good outcome (**C**), melanoma with and without local recurrence (**D**) and with or without systemic metastasis (**E**), respectively. *p < 0.05, ns: not significant (Mann–Whitney U test).

Cell type enrichment analysis using xCell^19^ revealed that numerous immune cell types were enriched in the tumor microenvironment (TME) of CM. Among them, plasmacytoid dendritic cells (pDC), natural killer T cells (NKT), B cells, activated dendritic cells (aDC) and mast cells were most significantly increased in CM compared to healthy conjunctiva (log2FC of enrichment scores between CM and control: 2.5, 2.3, 2.1, 1.4 and 1.3, respectively, *p* < 0.05, Fig. 3A). Furthermore, melanocytes and pericytes were significantly enriched in CM compared to control conjunctiva (log2FC: 1.6 and 1.8, respectively, *p* < 0.05). An overview of the enrichment scores of all 64 analyzed immune and stroma cell types is shown in Supplementary Fig. 2. The cell type analysis also demonstrated that melanoma and control samples clustered according to their histological diagnosis based on their cell type enrichment scores, indicating the significance of the TME in CM (Fig. 3A). This finding was unaffected when all 64 cell types were included in the analysis (data not shown). Immune enrichment scores (composite score of all analyzed immune cell types) were significantly higher in CM (mean: 0.063, SD: 0.057) when compared to healthy conjunctiva (mean: 0.019, SD: 0.020, *p* = 0.034), whereas stroma scores (composite score of all analyzed stroma cell types) did not significantly differ (mean: 0.028, SD: 0.023 and mean: 0.032, SD: 0.027, *p* = 0.847) (Fig. 3B). Next, we investigated whether immune and stroma scores differed between melanoma with poor and good clinical outcome and found that neither immune or stroma scores, nor the cell composition differed significantly when taking both clinical endpoints—metastasis and local recurrence—into account (Fig. 3A, C). While the immune scores between melanoma with and without local recurrence did not differ significantly (mean: 0.077, SD: 0.070 and mean: 0.053, SD: 0.050, *p* = 0.745), the immune scores in the two melanoma samples with systemic metastasis (mean: 0.012, SD: 0.002) were significantly lower compared to all other melanoma (mean: 0.074 SD 0.057, *p* = 0.041, (Fig. 3D, E). Stroma scores, in contrast, were not significantly different in metastasized or recurrent tumors (Fig. 3 D-E).

### Transcriptional characterization of conjunctival melanoma

**Figure 4:**
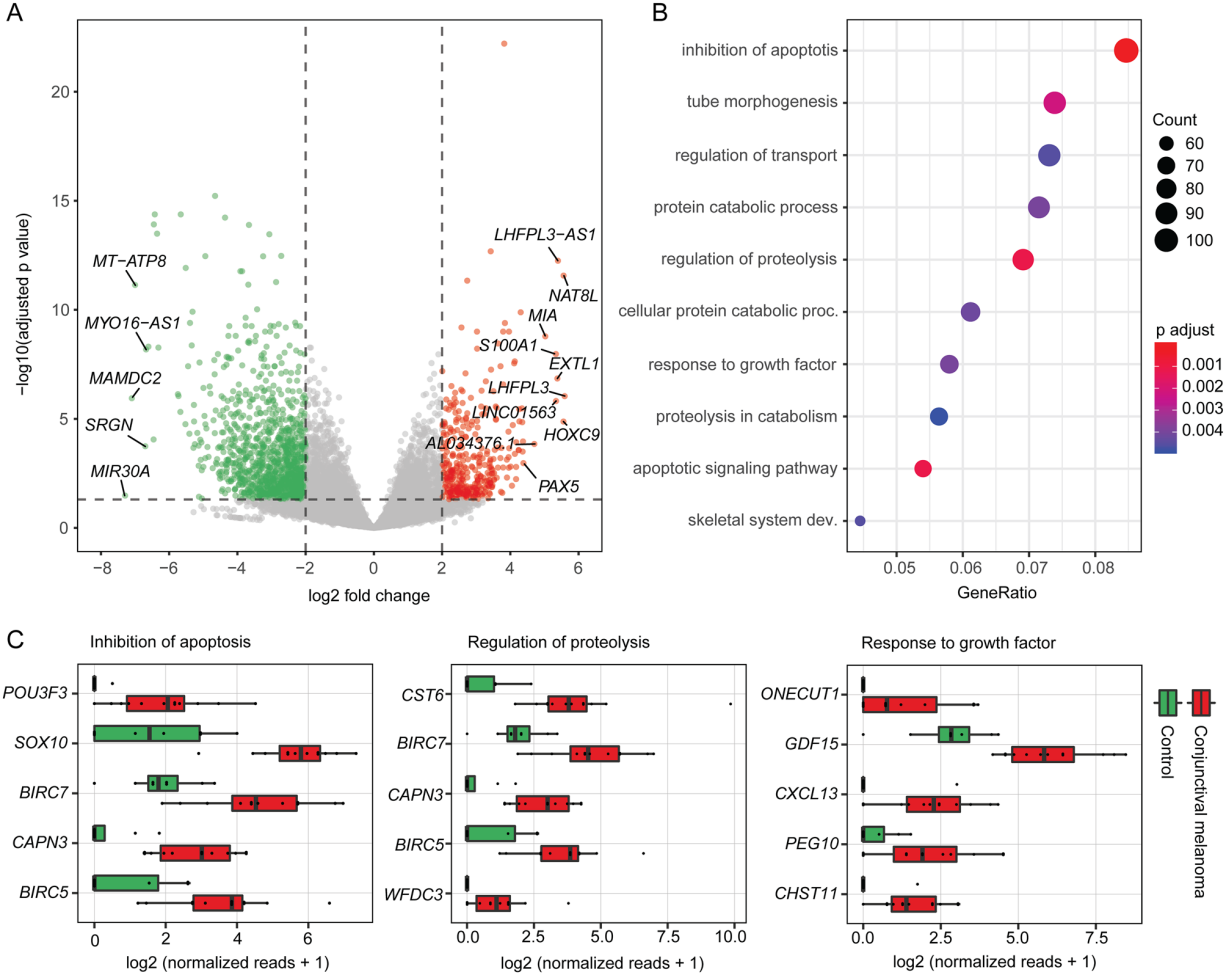
Characterization of the transcriptome of conjunctival melanoma (CM). (**A**) Volcano plot showing the up- and downregulated genes (red and green, respectively) in CM in comparison to normal conjunctiva. Genes not differentially expressed are shown in grey. The top ten upregulated as well as the top five downregulated genes in melanoma are labeled. (**B**) Gene ontology (GO) analysis of the differentially expressed genes in conjunctival melanoma. The top ten biological processes, which the DEG were involved in, are shown in the dot plot. “Inhibition of apoptosis” summarizes the two GO terms “negative regulation of apoptotic process” and “negative regulation of programmed cell death”, which overlapped with all 106 associated DEG. The size of the dots represents the number of associated genes (count). The adjusted *p* value of each GO term is shown by color. The gene ratio describes the ratio of the count to the number of all DEG. (**C**) Box plots illustrating normalized reads of the top five upregulated factors of three disease-relevant GO terms arranged by log2 fold change.

Differential gene expression analysis revealed 363 up- and 1096 downregulated genes in CM compared to healthy conjunctiva (Fig. 4A). Among them, *LHFPL3* (LHFPL tetraspan subfamily member 3), *NAT8L* (N-acetyltransferase 8 like), *HOXC9* (homeobox C9), *LHFPL3-AS1* (LHFPL3 antisense RNA 1) and *EXTL1* (exostosin like glycosyltransferase 1) were the five most significantly upregulated factors in CM (Fig. 4A). *MIR30A* (microRNA 30a), *MAMDC2* (MAM domain containing 2),* MT-ATP8* (mitochondrially encoded ATP synthase membrane subunit 8), *SRGN* (serglycin) and *MYO16-AS1* (MYO16 antisense RNA 1) were the top five downregulated genes in CM (Fig. 4A). Gene ontology (GO) analysis revealed, that the DEG contributed to biological processes such as inhibition of apoptosis (GO:0043066, GO:0043069), apoptotic signaling pathway (GO:0097190), proteolysis (GO:0030162, GO:0051603), tube morphogenesis (GO:0035239), protein catabolism (GO:0030163, GO:0044257), response to growth factor (GO:0071363) and transport of substances (GO:0051050) (Fig. 4B). A total of 106 DEG were associated to inhibition of apoptosis, among which 20 where up- and 86 were downregulated in CM (Supplementary Fig. 2). The top five upregulated genes in CM with regard to inhibition of apoptosis were *POU3F3* (POU class 3 homeobox 3),* SOX10* (SRY-box transcription factor 10),* BIRC7* (baculoviral IAP repeat containing 7),* CAPN3* (calpain 3) and *BIRC5* (baculoviral IAP repeat containing 5) (Fig. 4C). *CAPN3*, *BIRC5* and *7*, as well as *CST6* (cystatin E/M) and *WFDC3* (WAP four-disulfide core domain 3), also appeared among the top five genes playing a role in proteolysis (Fig. 4C). *ONECUT1* (one cut homeobox 1), *GDF15* (growth differentiation factor 15), *CXCL13* (C-X-C motif chemokine ligand 13), *PEG10* (paternally expressed 10) and *CHST11* (carbohydrate sulfotransferase 11) were the top five DEG in response to growth factor (Fig. 4C).

### Prognostic transcriptome signature of conjunctival melanoma

**Figure 5:**
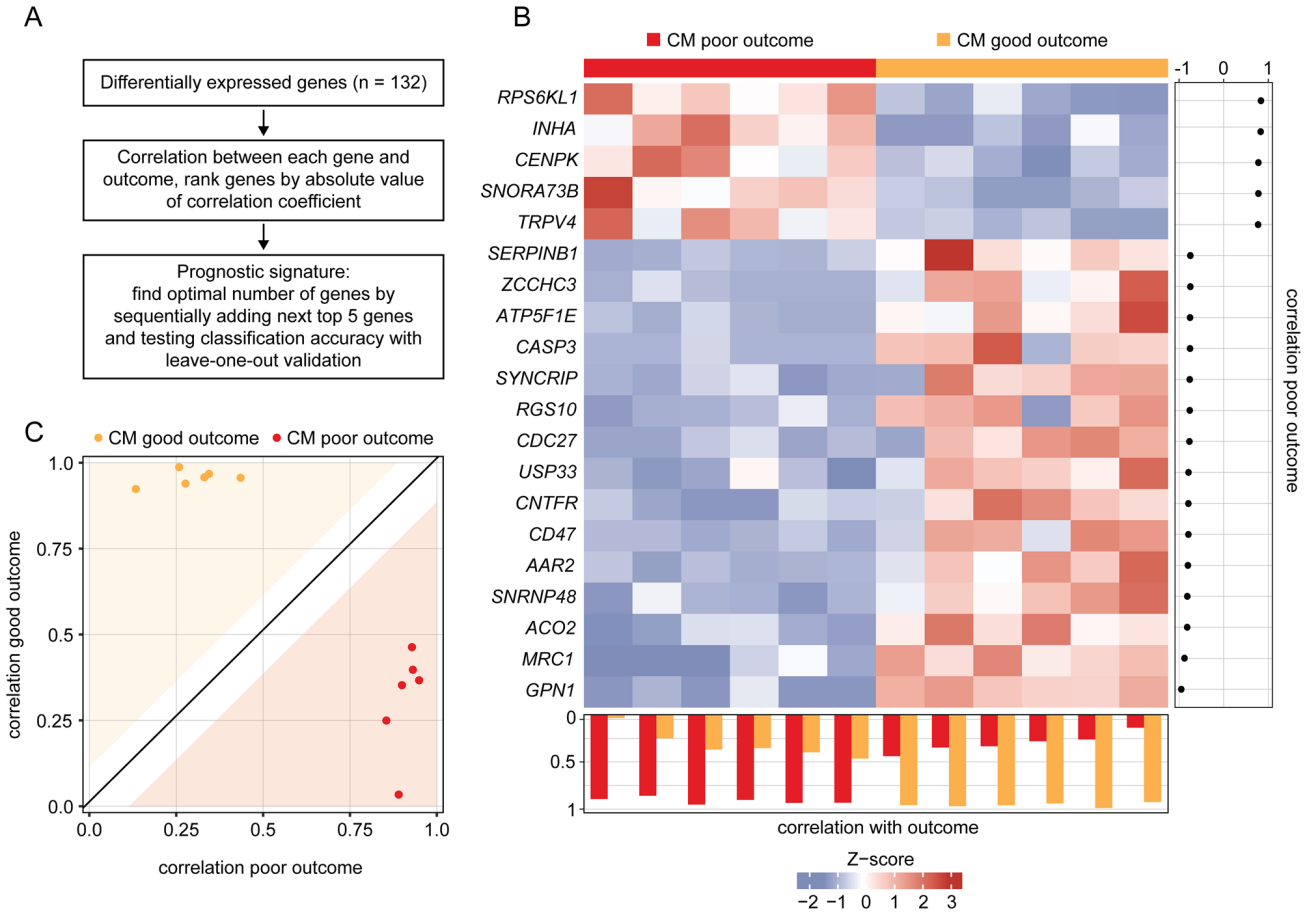
Prognostic transcriptome signature of conjunctival melanoma (CM). (**A**) Workflow of analysis. DEG between melanoma with poor and good clinical outcome were used to define a prognostic signature. Clinical outcome was defined based on the presence or absence of local recurrence and/or systemic metastases with a follow up of at least 24 months. (**B**) Heatmap of expression data of 20 prognostic marker genes of CM with good (orange) and poor outcome (red). Each row represents one gene and each column one tumor. The z-score represents a gene’s expression in relation to its mean expression by standard deviation units (red: upregulation, blue: downregulation). The genes are ordered according to their correlation with poor outcome, placing the gene with the highest correlation coefficient at the top (correlation coefficients are shown beside the heatmap). Tumors in the columns are ordered according to the correlation between the expression values of all 20 signature genes of one sample to the mean expression values of the other samples of both outcomes using leave-one-out validation (see methods). The correlation coefficients for both outcomes are shown below the heatmap and in (**C**): The white space around the diagonal is defined by the standard deviation of the distances of each sample from the diagonal. A sample that lies within the colored area can be assigned to an outcome with high probability.

Genes with prognostic relevance for CM were identified by determining the DEG between melanoma with poor and good clinical outcome in a first step (Fig. 5A). Poor clinical outcome was defined based on the presence or absence of local recurrence and/or systemic metastases, respectively, with a follow up of at least 24 months (mean follow-up: 7.1 years, min: 2.2, max: 16.7). In a second step, the correlation coefficient between each gene and outcome was calculated and the top genes were selected according to the absolute value of their correlation coefficient. By stepwise increasing the number of genes and testing the classification accuracy using ROC and leave-one-out validation (see methods for details), it became evident that a gene signature of twenty genes had the optimal accuracy for classifying clinical outcome in CM (AUC = 1.0). The expression profile of these twenty genes in all 12 melanoma samples is shown in the heatmap in Fig. 5B, with the genes and samples arranged according to their correlation with clinical outcome (Fig. 5B, description and right and bottom panel). Five prognostic genes were identified, which were upregulated in CM with poor outcome: *RPS6KL1* (ribosomal protein S6 kinase like 1), *INHA* (inhibin subunit alpha), *CENPK* (centromere protein K), *SNORA73B* (small nucleolar RNA, H/ACA box 73B) and *TRPV4* (transient receptor potential cation channel subfamily V member 4). The top five of 15 downregulated genes in CM with poor outcome were *GPN1* (GPN-loop GTPase 1), *MRC1* (mannose receptor C-type 1), *ACO2* (aconitase 2), *SNRNP48* (small nuclear ribonucleoprotein U11/U12 subunit 48) and *AAR2* (AAR2 splicing factor) (Fig. 5B). The classification of poor and good outcome based on the twenty signature genes is shown in Fig. 5C (see methods for details). GO analysis revealed, that the twenty signature genes were mainly involved in biological processes such as cell proliferation, apoptosis and several immune processes as illustrated in the cnetplot in Supplementary Fig. 3.

Finally, we investigated whether clinical or histopathological parameters, such as age, gender, pT category, tumor thickness, localization of the CM or tumor pigmentation, were associated with clinical outcome (Table 2). Uni- and multivariate logistic regression analysis revealed that the clinical and histopathological parameters assessed were not significantly associated with outcome, whereas the transcriptome signature remained significant in multivariate analysis (*p* < 0.001).

**Table 2:** Multivariate analysis of clinical and histopathological factors regarding the clinical outcome of conjunctival melanoma.

	Poor prognosis	Good prognosis	*p*
n	6	6	–
Age at surgery (years)	57.7 (37.1–85.3)	60.1 (27.3–81.0)	0.818
Sex			> 0.999
Male	2 (33.3%)	1 (16.6%)	
Female	4 (66.6%)	5 (83.3%)	
Skin color			> 0.999
White	6 (100.0%)	6 (100.0%)	
pT-category			0.135
pT1a	3 (50%)	6 (100%)	
pT1b	1 (16.7%)	0 (0%)	
pT3b	2 (33.3%)	0 (0%)	
Tumor-thickness (µm)	1201.0 (376.0–2720.0)	687.2 (199.0–1002.0)	0.630
Localization			0.262
Bulbar conjunctiva	3 (50%)	6 (100%)	
Fornix	1 (16.7%)	0 (0%)	
Tarsal conjunctiva	1 (16.7%)	0 (0%)	
Limbus	1 (16.7%)	0 (0%)	
Pigmentation			0.439
Melanotic	6 (100.0%)	4 (66.7%)	
Amelanotic	0 (0.0%)	2 (33.3%)	
Transcriptome signature			< 0.001
Classified: poor	6 (100.0%)	0 (0.0%)	
Classified: good	0 (0.0%)	6 (100.0%)	

pT-classification according to the eighth edition of the AJCC conjunctival melanoma staging system^18^. Data is shown as mean (range) or as absolute (relative) numbers.

## Discussion

Gene expression analysis provides important insights into the molecular mechanisms of tumorigenesis and -progression and has helped to define new therapeutic targets in numerous malignancies. However, the transcriptional analysis of rare tumors, such as CM, has so far been hampered by their low incidence, challenging a prospective analysis of fresh tissue. 3′-RNA sequencing methods, such as the Massive Analysis of cDNA Ends (MACE), allow transcriptome analysis of FFPE samples^20^, in which RNA degradation predominantly occurs at the 5′ end^21^. The present study uses MACE RNA sequencing of FFPE samples to characterize the cellular tumor microenvironment (TME) and the transcriptional profile of CM compared to healthy conjunctiva and identifies a prognostic transcriptome signature that allows the classification of poor and good clinical outcome for CM.

The cellular tumor microenvironment is known to modulate tumorigenesis, tumor progression, therapeutic response and clinical outcome of various malignancies^22^. Transcriptome-based cell type enrichment analysis using xCell^19^ revealed that the tumor microenvironment of CM was predominantly characterized by an enrichment of numerous immune cell types, including plasmacytoid dendritic cells (pDC), natural killer T cells (NKT), B cells and mast cells. pDCs and NKTs are known to be important components of the TME in various malignancies and are essentially involved in the regulation of anti-tumor immunity^23,24^. There is also evidence of tumor-resident B-cells in skin melanoma, which are associated with a favorable prognosis, suggesting an involvement in anti-tumor immunity and highlighting their potential as a therapeutic target^25^. Finally, mast cell infiltration has been observed in various tumors, including malignant cutaneous melanoma, breast and colorectal cancer^26^. Mast cells are able to modulate the TME by releasing different mediators, including a variety of proangiogenic factors and several matrix metalloproteinases that can increase the invasiveness of the tumor^26^. Taken together, our results suggest that the enriched immune cells might play a crucial role in the development of CM and thus may represent potential therapeutic targets for a specific anti-tumor immune response. Additionally, the present study revealed that CM with systemic metastases had significantly lower immune scores than non-metastatic tumors, a finding that has previously been reported for different cancer types^27,28^. In a study of Mlecnik et al.^27^, a low immune score was associated with an increased risk of systematic metastases in patients with colorectal cancer. The authors conclude that patients in early stages could benefit most from checkpoint T-cell therapies to prevent the development of distant metastases because these tumors have higher immune scores^27^. In CM, the immune analysis could therefore be a promising diagnostic approach to predict the development of systemic metastases and might also be used to predict the response to immune therapy, for which, in contrast to other tumor localizations, a local application could also be considered. However, it is important to note that of the twelve CM included, only two were associated with metastasis, which requires further investigation to validate their diagnostic and therapeutic potential.

The transcriptional signature of CM provided in this study differed significantly from healthy conjunctival samples and revealed 1459 differentially expressed genes (DEG). Gene ontology (GO) analysis demonstrated that these DEG were mainly involved in biological processes such as inhibition of apoptosis, apoptotic signaling pathway, proteolysis and response to growth factor. Among the apoptosis inhibitors, *POU3F3* was the top upregulated DEG in CM. This factor and especially the long non coding (lnc) RNA *linc-POU3F3*, which was also among the upregulated DEG, are known to play a role in tumor cell proliferation, inhibition of apoptosis, as well as in angiogenesis in several malignancies^29–35^. siRNA-mediated knockdown of linc-*POU3F3* reduces tumor cell proliferation and invasion and increase apoptosis in colorectal cancer cells^33^ and may therefore represent a therapeutic approach for the treatment of CM. Another top expressed factor in inhibition of apoptosis was *SOX10*, a nuclear transcription factor that is involved in the differentiation of neural crest progenitor cells to melanocytes and is known as a sensitive and specific marker for skin melanoma^36^, that has not yet been described in CM. In skin melanoma cells, *SOX10* mediates the invasion through *MIA* (*Melanoma Inhibitory Activity*)^37^, which was also among the top upregulated DEG in CM. In addition, *SOX10* was recently identified as an oncogene in skin melanoma that could be inhibited by the miRNA miR-31^38^. *BIRC5* and *BIRC7* were among the top 5 DEG associated with inhibition of apoptosis and regulation of proteolysis. Both factors belong to the "inhibitors of apoptosis proteins" family and are upregulated in uveal melanoma as well^39^. The most significantly upregulated DEG in CM was *LHFPL3*, which is also highly expressed in malignant glioma and can be inhibited by *miRNA-218-5p* thus reducing the invasiveness of glioma cells^40^. Further studies will be necessary to investigate the presented factors and signaling pathways in the development of CM in more detail and to validate them as potential therapeutic targets for the treatment of CM.

In search of a prognostic tool that provides information on the aggressiveness of CM in relation to local recurrence or systemic metastases, this study identified a prognostic transcriptional signature comprised of twenty genes allowing a classification of the clinical outcome of CM with high accuracy. The clinical and histopathological standard predictors, such as TNM stage, tumor thickness and tumor localization^6^ were not significantly associated with the clinical outcome in our study, although some tendency was observed which may be explained by the low sample size of our study. These results underline the potential of a transcriptional analysis to categorize clinical outcome. While most of the genes predicting outcome of CM have already been described as prognostic factors in other malignancies, such as *CENPK*^41–46^, *INHA*^47–50^, *RPS6KL1*^51^, *CASP3* (*Caspase 3*)^52^, *SERPINB1* (*Serpin Family B Member 1*)^53^, *USP33* (*Ubiquitin Specific Peptidase 33*)^54–59^, *TRPV4*^60^ and *ACO2*^61^, some of the predicting factors have not yet been associated to cancer prognosis, such as *GPN1*, *SNRNP48*, *AAR2* and *SNORA73B*. Among the mentioned factors, *GPN1* was the gene with the highest correlation coefficient with good clinical outcome in CM. *GPN1* is involved in the nuclear translocation of *XPA* (*XPA*,* DNA Damage Recognition And Repair Factor*), an important factor that controls nucleotide excision repair^62^, and has been linked to the development of oral cancer in a genome-wide association study^63^. RNA splicing regulators such as *AAR2* and *SNRNP48*, on the other hand, are emerging as a new class of oncoproteins and tumor suppressors^64^, and small nucleolar RNAs such as *SNORA73B* seem to play important roles in tumorigenesis^65^. However, none of the aforementioned factors has been linked to cancer prognosis and further studies are warranted to validate their prognostic value in CM and other malignancies.

We acknowledge that this study is limited by its retrospective single center design and its relatively small sample size. However, a prospective single center study does not appear feasible due to the very low incidence of CM. A prospective multicenter study would be necessary to overcome these limitations. Another limitation is the lack of external validation of the prognostic transcription signature, since no sequencing data of CM are available so far. Instead, we used the concept of leave-one-out validation, as previously described^66,67^. Furthermore, in contrast to single cell RNA sequencing (scRNA), bulk RNA sequencing cannot provide insights into cell heterogeneity and thus cannot reveal cell-specific transcriptional profiles to discern possible subtypes of tumor cells. However, scRNA sequencing is not feasible on FFPE samples. Therefore, we employed a bulk RNA sequencing-based cell type enrichment analysis using xCell^19^ which is one of the most accurate tools available^68^ to characterize the cell types involved in the tumor microenvironment of CM. It is important to emphasize that the cell type investigations are based on in silico analysis and have not been validated histologically due to the limited number of specimens. This needs to be considered in future studies.

In summary, the present study provides new insights into the cellular tumor microenvironment and the transcriptional profile of CM. It adds new prognostic biomarkers and diagnostic tools which can help to improve identification of high-risk patients and may lead to new options of targeted therapy for CM.

## Methods

### Patients and clinical outcome

A total of 12 CM samples from 12 patients who underwent tumor resection at the Eye Center of the University of Freiburg between 1996 and 2017 were retrospectively included in this study. Six cases each with good or poor clinical outcome with a follow-up of at least 24 months were examined, the latter being defined by local recurrence and/or systemic metastases. Eight healthy conjunctival samples from eight patients who underwent retinal detachment surgery between 2013 and 2016 served as controls. All methods were carried out in accordance with relevant guidelines and regulations and informed consent was obtained from all subjects. Ethics approval was granted from Ethics Committee of the Albert-Ludwigs-University Freiburg (approval number 481/19).

### Formalin fixation and paraffin embedding

Formalin fixation and paraffin embedding (FFPE) of tissue samples was performed immediately after surgery according to routine protocols, as previously described^69,70^. Briefly, samples were fixed immediately after surgery in 4% formalin for 12 h, dehydrated in alcohol and processed for paraffin embedding. Histological diagnoses were made by an experienced ophthalmic pathologist (CAH). Hematoxylin and eosin stained slides were imaged using a Hamamatsu NanoZoomer S60 (Hamamatsu Photonics, Herrsching, Germany).

### RNA isolation

After melting the block, tumor-free tissue areas were removed and the tumor, as well as the control FFPE samples were stored in tubes until RNA isolation, which was performed as previously described^20,70^. Briefly, total RNA was isolated from FFPE samples using the Quick-RNA FFPE Kit (Zymo Research). Following a DNAse I digestion using the Baseline-ZERO kit (Epicentre), the RNA concentration was measured with the Qubit RNA HS Assay Kit on a Qubit Fluorometer (Life Technologies). The RNA quality was determined with the RNA Pico Sensitivity Assay on a LabChip GXII Touch (PerkinElmer).

### RNA sequencing

RNA sequencing was performed using massive analysis of cDNA ends (MACE), a 3′ RNA sequencing method, as previously described^20,70^. We recently demonstrated that MACE allows sequencing of FFPE samples with high accuracy^71^. Briefly, 20 barcoded libraries comprising unique molecule identifiers were sequenced on the NextSeq 500 (Illumina) with 1 × 75 bp. PCR bias was removed using unique molecular identifiers.

### Bioinformatics

Sequencing data (fastq files) were uploaded to and analyzed on the Galaxy web platform (usegalaxy.eu)^72^, as previously described^73^. Quality control was performed with *FastQC Galaxy Version 0.72* (https://www.bioinformatics.babraham.ac.uk/projects/fastqc/ last access on 11/19/2019)*.* Reads were mapped to the human reference genome (Gencode, release 32, hg38) with *RNA STAR Galaxy Version 2.7.2b*^74^ with default parameters using the Gencode annotation file (Gencode, release 32, https://www.gencodegenes.org/human/releases.html). Reads mapped to the human reference genome were counted using *featureCounts Galaxy Version 1.6.4*^75^ with default parameters using the aforementioned annotation file. The output of featureCounts was imported to RStudio (Version 1.2.1335, R Version 3.5.3). Gene symbols and gene types were determined based on ENSEMBL release 98 (Human genes, GRCh38.p12, download on 11/19/2019)^76^. Genes without EntrezID and with zero mean reads were removed from analysis. Principal component analysis (PCA)^77^ was applied to check for potential batch effects, which were removed by the *limma* function *removeBatchEffect*^78^ and by consideration within the linear model of DESeq2^77^. Differential gene expression was analyzed using the R package DESeq2 Version 1.22.2^77^ with default parameters (Benjamini–Hochberg adjusted *p* values). Transcripts with log2 fold change (log2 FC) > 2 or < -2 and adjusted *p* value < 0.05 were considered as differentially expressed genes (DEG). Heatmaps were created with the R package *ComplexHeatmap 1.20.0*^79^. Other data visualization was performed using the *ggplot2* package^80^. Gene enrichment analysis and its visualization were done using the R package *clusterProfiler 3.10.1*^81^. Cell type enrichment analysis was performed using xCell^19^. The tool uses sequencing-derived transcriptomic signatures of 64 distinct immune and stroma cell types to estimate the relative contributions of these cells to a bulk RNA transcriptome. Transcripts per million were calculated as an input for the analysis based on the output of *featureCounts* (assigned reads and feature length), as previously described^82^. xCell enrichment scores were compared between different groups using the Mann–Whitney U test. The log2 fold change of enrichment scores between different groups was defined as the log2 of the quotient of mean enrichment scores of each group. To define a prognostic transcriptome signature of CM, a three-step method, as described by van´t Veer et al.^66^, was applied. In a first step, DEG between CM with poor and good outcome were determined, as described above. Subsequently, the Pearson correlation between each gene and outcome was calculated. All genes were then arranged by the absolute value of their correlation coefficient. The top five genes were selected and their expression profile in one sample was correlated to each gene’s mean expression of the remaining samples of the poor and good prognosis group, respectively. These steps were repeated until each sample was left out once (leave-one-out validation), as described previously^66,67^. Classification accuracy was determined by calculating the area under the curve (AUC) of receiver operating curves (ROC). To find the optimal number of prognostic genes, the next top five genes were added to the signature and its classification accuracy was evaluated, as described above. This process was repeated until the AUC stopped improving.

## Supplementary information

Supplementary file1

## Data Availability

The sequencing data are available in the Gene Expression Omnibus Database under the accession number GSE148387.
