# Supplementary material for: Transcriptional characterization of conjunctival melanoma identifies the cellular tumor microenvironment and prognostic gene signatures
Source: Sci Rep. 2020 Oct 12;10:17022. doi: 10.1038/s41598-020-72864-0 (PMC7550331; doi:10.1038/s41598-020-72864-0)
Supplement: Supplementary file 1 — Supplementary file1 [file 41598_2020_72864_MOESM1_ESM.pdf]

SUPPLEMENT

Transcriptional Characterization of Conjunctival Melanoma Identifies the Cellular Tumor Microenvironment and Prognostic Gene Signatures

Julian Wolf[1], Claudia Auw-Haedrich[1], Anja Schlecht[1], Stefaniya Boneva[1], Hans Mittelviefhaus[1], Thabo Lapp[1], Hansjürgen Agostini[1], Thomas Reinhard[1], Günther Schlunck[1], and Clemens AK Lange[1*]

[1] Eye Center, Medical Center, Faculty of Medicine, University of Freiburg, Germany

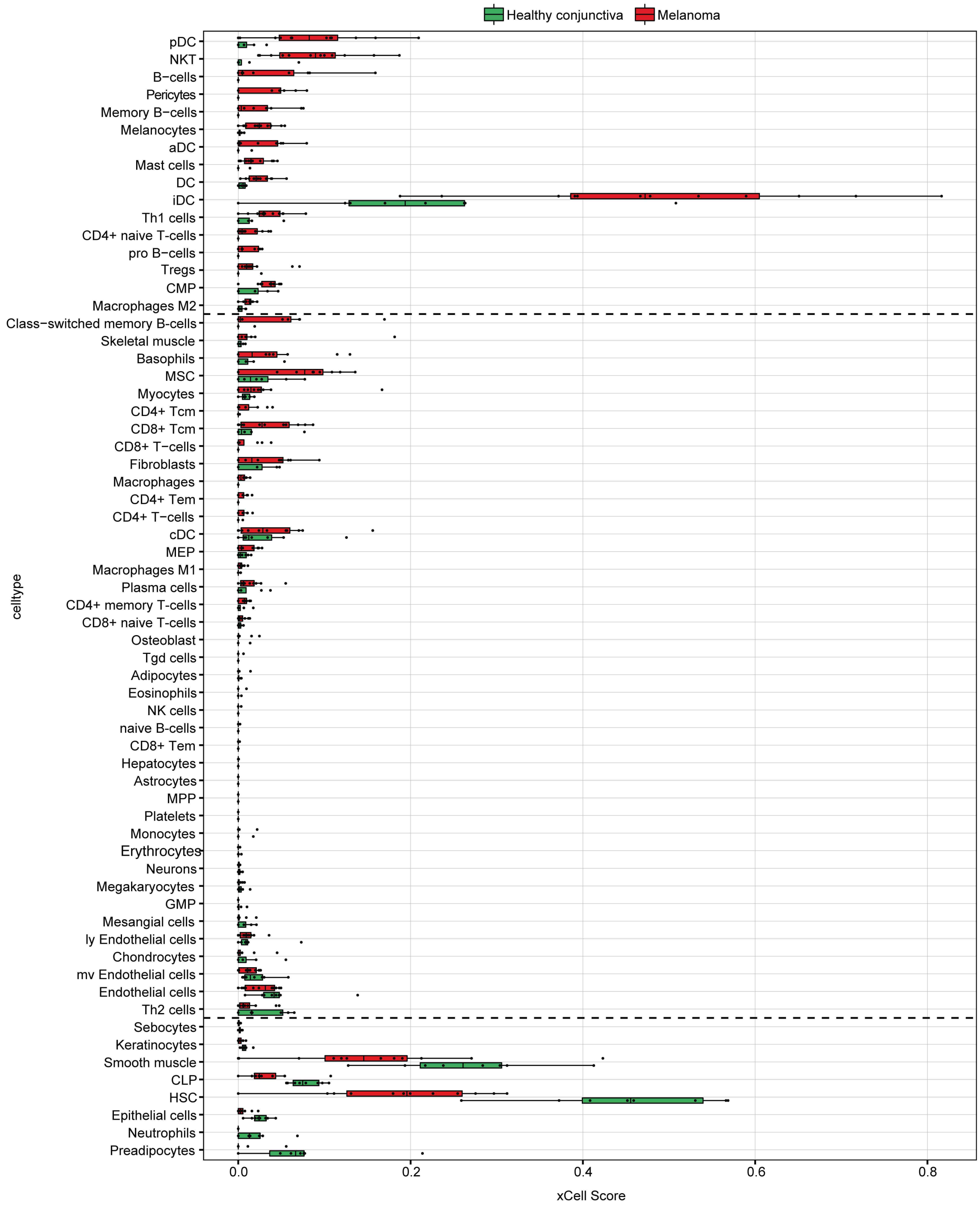

Supplement Figure 1: Boxplots illustrating the xCell enrichment scores of all 64 analyzed immune and stroma cell types in conjunctival melanoma compared to healthy conjunctiva. Each dot represents one sample. Cell types, which were positively enriched in melanoma, are presented at the top, above the upper dashed line, whereas negatively enriched cell types are shown below the lower dashed line. Cell types, which were not significantly different between melanoma

and control samples, are shown in between. pDC: plasmacytoid dendritic cell, NKT: natural killer cells, aDC: activated dendritic cell, DC: dendritic cell, iDC: immature dendritic cells, Th1: type 1 T-helper cells, Tregs: regulatory T cells, CMP: common myeloid progenitor, MSC: mesenchymal stem cells, Tcm: central memory T cell, Tem: effector memory T cell, cDC: classical DC, MEP: megakaryocyte–erythroid progenitor, tgd cells: T gamma delta cells, NK cells: natural killer cells, MPP: multipotent progenitor, GMP: granulocyte-monocyte progenitor, ly: lymphatic, mv: microvascular, Th2: type 2 T-helper cells, CLP: common lymphoid progenitor, HSC: hematopoietic stem cell.

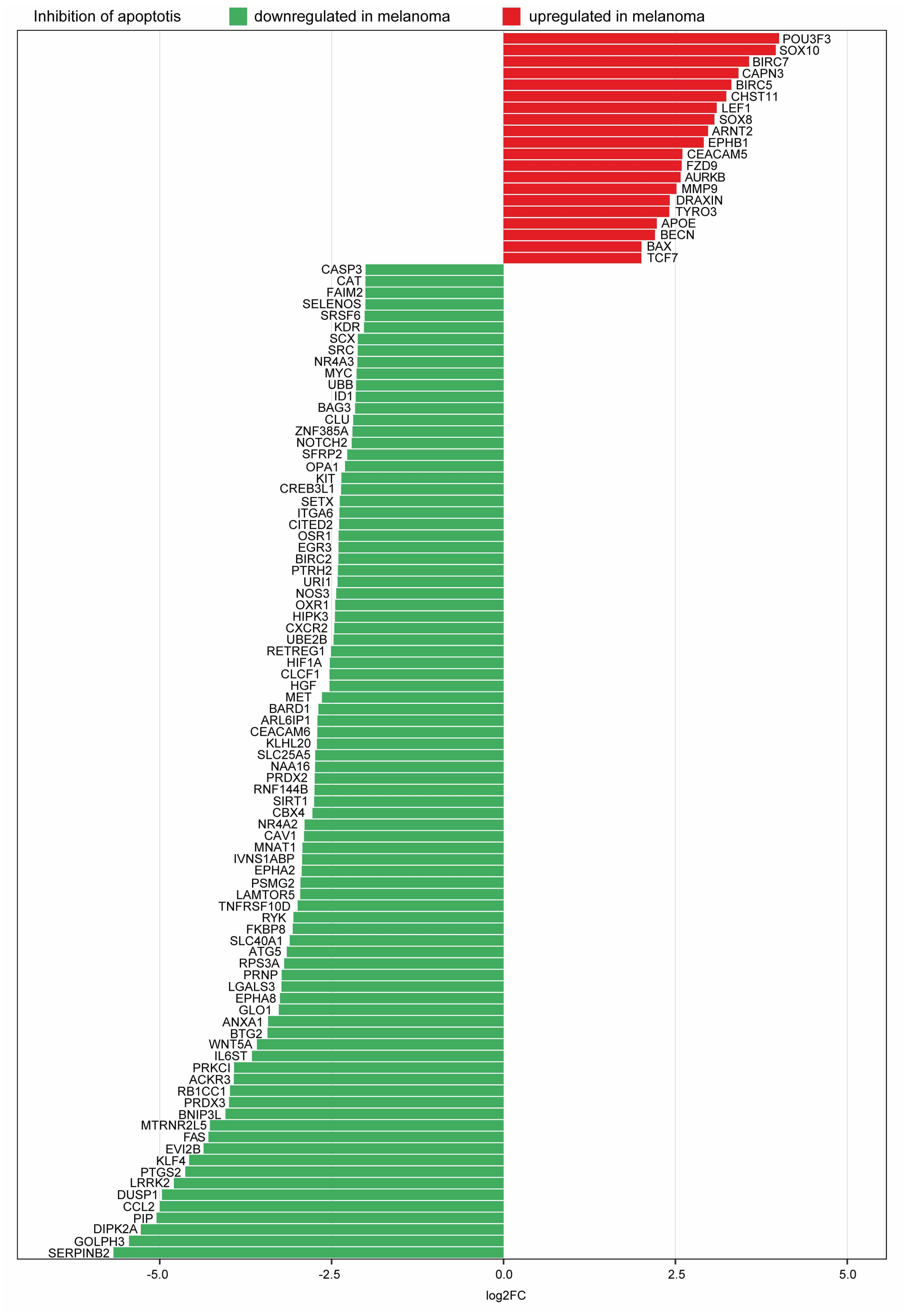

Supplement Figure 2: Barplot of all 106 DEG between conjunctival melanoma and normal conjunctiva associated with inhibition of apoptosis. Inhibition

of apoptosis summarizes the two GO terms “negative regulation of apoptotic process” and “negative regulation of programmed cell death”, which overlapped with all 106 associated DEGs.

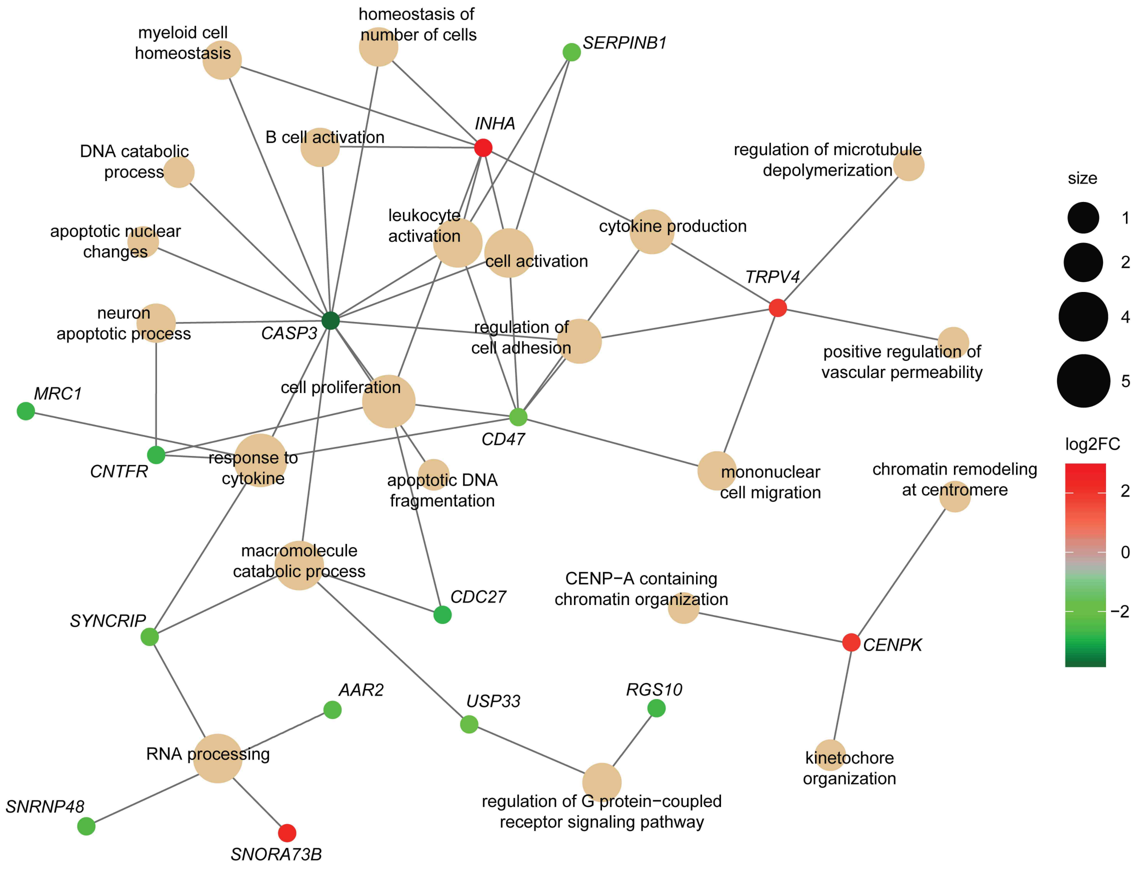

Supplement Figure 3: Cnetplot illustrating the biological processes (large circles), which the signa-ture genes (small circles) are involved in (Gene Ontology (GO)). Size: number of genes associated with one GO term, color: log2 fold change between poor (red) and good outcome (green).
